# Vocational training of health mediators of primary care teams improves their sense of coherence

**DOI:** 10.1093/eurpub/ckac131.037

**Published:** 2022-10-25

**Authors:** K Kósa, C Katona, É Bíró, S Vincze

**Affiliations:** 1 Department of Behavioral Sciences, Faculty of Medicine, University of Debrecen, Debrecen, Hungary; 2 Department of Preventive Medicine, University of Debrecen, Debrecen, Hungary; 3 Department of Economics and Methodology, University of Debrecen, Debrecen, Hungary

## Abstract

**Background:**

A Primary Care Model Programme had been implemented in Hungary between 2013-2017 in which group practices were established that employed - among others - nonprofessional health workers (health mediators, similar to community health workers) to facilitate access for the most disadvantaged population groups. The health of mediators, themselves mostly disadvantaged ethnic Roma, was monitored every odd year of the Programme.

**Methods:**

A repeated cross-sectional health interview survey had been implemented inviting all health mediators who were employed at the time of the survey. The same questionnaire was used in all 3 surveys with items from the European Health Interview Survey 2009 and validated versions of other scales.

**Results:**

Positive changes occurred in the health status of mediators during 5 years of follow-up. Significant improvement in mental health occurred among those who completed on-the-job vocational training. By 2017, significant increase in sense of coherence was observed among those who obtained vocational qualification as opposed to those who did not. The proportion of highly stressed mediators showed a significant increase among those with no vocational training. Improvement was detected in all mediators in health awareness, dysfunctional attitudes, psychological stress and smoking prevalence.

**Conclusions:**

Significant improvement in mental status among those who obtained on-the-job vocational qualification were observed during follow-up of ethnic Roma health mediators in the programme in which they were equal members of the primary health care team. Employment of health mediators in primary care teams not only contributed to improving access to care for disadvantaged groups but also improved the mental health of mediators themselves.

**Key messages:**

• Nonprofessional health mediators are important members of primary care teams servicing disadvantaged populations.

• Significant improvement in mental status during 5 years of follow-up occurred among Roma health mediators who received vocational training and were equal members of primary health care teams.

